# Natural history study and statistical modeling of disease progression in a preclinical model of myotubular myopathy

**DOI:** 10.1242/dmm.049284

**Published:** 2022-07-25

**Authors:** Suzie Buono, Arnaud Monseur, Alexia Menuet, Anne Robé, Catherine Koch, Jocelyn Laporte, Leen Thielemans, Marion Depla, Belinda S. Cowling

**Affiliations:** 1 Dynacure, Illkirch 67400, France; 2 Pharmalex, Mont-St-Guibert 1435, Belgium; 3 Institut de Génétique et de Biologie Moléculaire et Cellulaire, Institut National de la Santé et de la Recherche Médicale U1258, Centre National de la Recherche Scientifique UMR7104, Université de Strasbourg, 67404 Illkirch, France; 4 2 Bridge, Zoersel 2980, Belgium

**Keywords:** Centronuclear myopathy, Myotubular myopathy, Preclinical disease model, Dynamin, Antisense oligonucleotide, Therapy, Neuromuscular disorder

## Abstract

Generating reliable preclinical data in animal models of disease is essential in therapy development. Here, we performed statistical analysis and joint longitudinal–survival modeling of the progressive phenotype observed in *Mtm1*^−/y^ mice, a reliable model for myotubular myopathy. Analysis of historical data was used to generate a model for phenotype progression, which was then confirmed with phenotypic data from a new colony of mice derived via *in vitro* fertilization in an independent animal house, highlighting the reproducibility of disease phenotype in *Mtm1*^−/y^ mice. These combined data were used to refine the phenotypic parameters analyzed in these mice and improve the model generated for expected disease progression. The disease progression model was then used to test the therapeutic efficacy of *Dnm2* targeting. *Dnm2* reduction by antisense oligonucleotides blocked or postponed disease development, and resulted in a significant dose-dependent improvement outside the expected disease progression in untreated *Mtm1*^−/y^ mice. This provides an example of optimizing disease analysis and testing therapeutic efficacy in a preclinical model, which can be applied by scientists testing therapeutic approaches using neuromuscular disease models in different laboratories.

This article has an associated First Person interview with the joint first authors of the paper.

## Abstract (precis)

**Summary:** This study optimized disease severity analysis and modeled disease progression in *Mtm1*^−/y^ mice, and confirmed this model using therapeutic *Dnm2* reduction in a dose–response analysis.

## INTRODUCTION

In a research setting, mouse models of disease provide an excellent tool to investigate disease pathophysiology and test therapeutic approaches. Mouse lines that recapitulate the disease phenotype can be used to identify novel therapeutic targets, and map in a temporal and dose-dependent manner the response to treatment. Animal models may also be useful in identifying and testing diagnostic, prognostic or therapeutic biomarkers. Pathological phenotypes in animals may be compared with the human disease, to help gain insight into the underlying pathophysiology that is relevant for patients, and to test the potential of various therapeutic modalities targeting the mechanism of action, including small molecules (chemically derived) and various biologics (e.g. gene and cell therapies).

Many potential therapies for neuromuscular diseases have moved from proof-of-concept in animal studies towards clinical trials in the past decade (Cowling and Thielemans, 2019). Often, proof-of-concept is generated using preclinical animal models of neuromuscular disease; however, translating preclinical data to a clinical setting is often challenging. Mouse models of human disease have limitations, which may be linked to the relatively uniform genetic background of animal cohorts compared to humans, the potentially complex genetic involvement in inherited neuromuscular disorders (causative gene and epistatic mutations) and the targeting of pathogenic pathways, which might not be similar between species. Considering these limitations is important when interpreting animal data. Optimizing the generation of relevant, reliable and reproducible preclinical data on disease phenotype and therapeutic potential is of high importance. Natural history studies in mice are a useful way to understand disease progression and standardize phenotyping parameters across studies. This has been performed in recent years in mdx mice, a frequently used mouse line to investigate Duchenne muscular dystrophy (van Putten et al., 2019; Gordish-Dressman et al., 2018).

In this study, we focus on a preclinical animal model for myotubular myopathy. Myotubular myopathy is a non-dystrophic, debilitating rare congenital disease, associated with muscle weakness and abnormally located nuclei and other organelles in skeletal muscle (Ravenscroft et al., 2015). Myotubular myopathy [also called X-linked centronuclear myopathy (CNM), XLCNM, XLMTM; OMIM 310400] is due to mutations in the phosphoinositide phosphatase myotubularin (MTM1) (Laporte et al., 1996). There are an estimated 2650 living patients with myotubular myopathy, in the USA, EU, Japan and Australia (Vandersmissen et al., 2018). *Mtm1*^−/y^ mice recapitulate myotubular myopathy; as observed in patients, mice display a severe myopathic phenotype and a reduced lifespan (Buj-Bello et al., 2002). This model has been used across laboratories from several continents, and has been used to provide the therapeutic proof-of-concept and supportive data contributing to the rationale for initiating several clinical trials (NCT04915846, NCT03199469, NCT04033159), thus highlighting the importance of data generated from this model. Previous studies suggested that increased DNM2 was largely responsible for the CNM phenotype observed in mice and patients (Cowling et al., 2014, 2011; Liu et al., 2011; Massana Munoz et al., 2019). Reduction of DNM2 was shown to rescue myotubular myopathy in mice (*Mtm1*^−/y^ mice) by genetic cross (Cowling et al., 2014) or systemic delivery of antisense oligonucleotides (ASOs) (Tasfaout et al., 2017; Koch et al., 2020), or by reducing DNM2 using an adeno-associated virus-mediated shRNA approach targeting *Dnm2* (Tasfaout et al., 2018).

The goal of this study was to describe disease progression and the variability with which it can occur, and to inform which phenotypic parameters are sufficient to predict disease progression in *Mtm1*^−/y^ mice. We performed statistical analysis and modeling of the progressive phenotype observed in *Mtm1*^−/y^ mice from retrospective (‘training’) and prospective (‘test’) data generated in *Mtm1*^−/y^ mice. These combined data were then used to improve both the phenotyping analysis performed in these mice and the model generated for normal disease progression. Finally, this model was tested and validated by performing a therapeutic dose–response study targeting *Dnm2*.

## RESULTS

### Natural history study analysis and model generation from training cohort

**Fig. 1.**
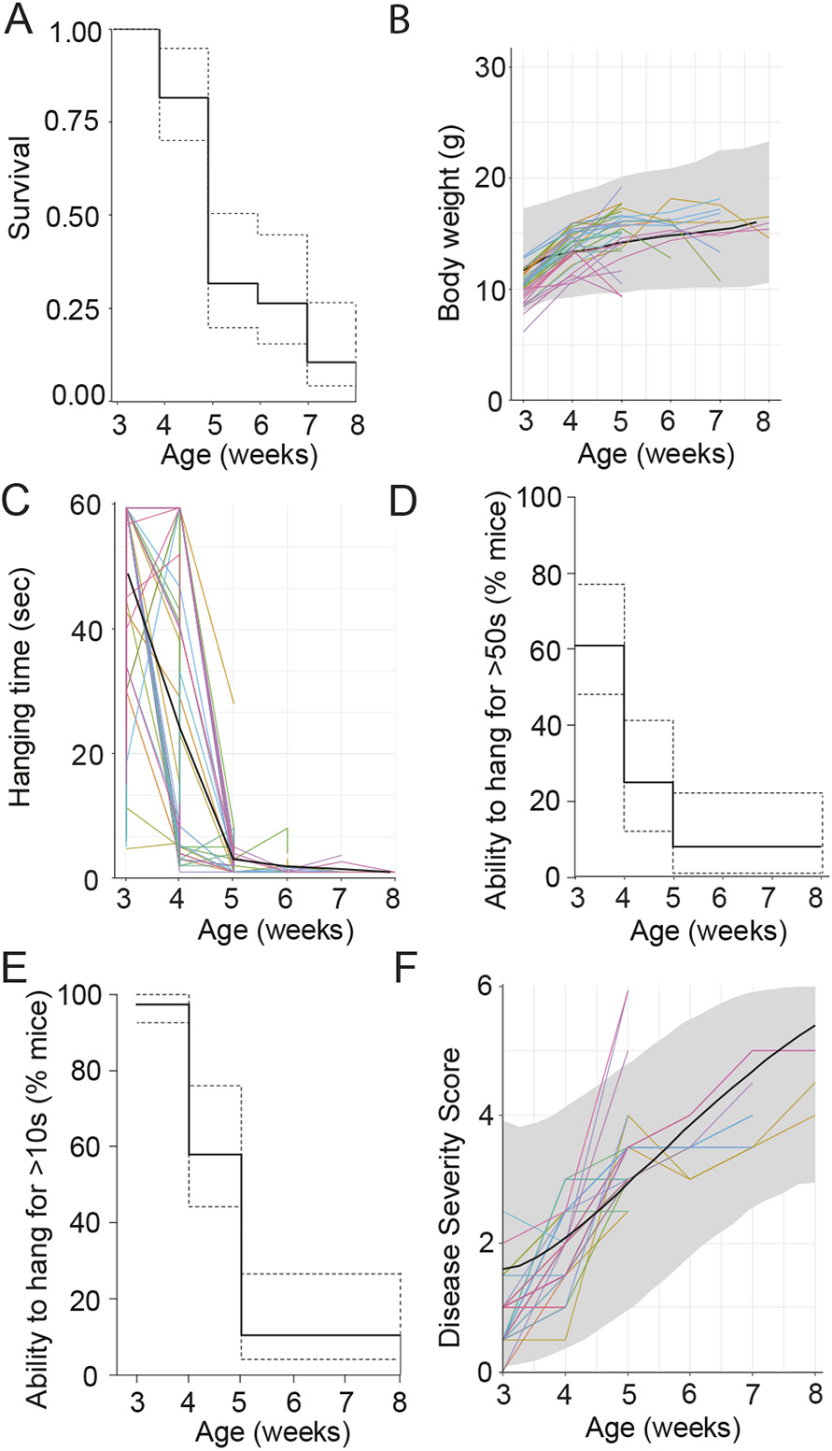
**Natural history study analysis and model generation from training cohort.** (A) Survival of mice, line of best fit (solid line) and 95% confidence interval (CI) (dashed lines) are shown. (B) Body weight (g) progression weekly in *Mtm1*^−/y^ mice. Individual mice (colored lines), line of best fit (black line) and prediction interval (shaded gray zone) are highlighted on the graph. (C) Hanging time (60 s maximum), with individual mouse progression (colored lines) and average (black line) shown. (D,E) Time to event analysis of hanging test, with cutoff times of 50 s (D) and 10 s (E) displayed. Graphs represent line of best fit (solid line) ±95% CI (dashed lines). (F) Disease severity score (DSS), with a score between 0 (unaffected) and 6 (most severely affected) awarded per mouse per week, based on six different phenotypes (see Table 1 for details). Modeling was performed to identify the line of best fit (black line) and prediction intervals (gray shaded area) for expected disease progression in *Mtm1*^−/y^ mice. All data in this figure represent analysis of *Mtm1*^−/y^ mouse phenotypes from the training cohort, starting from 3 weeks of age; individual mice represented as colored lines. *n*=38 male mice.

To generate a statistical model of disease progression in myotubular myopathy mice, we first focused on performing an analysis of previous natural history data generated from *Mtm1*^−/y^ mice across several studies (Koch et al., 2020), referred to as the ‘training cohort’. Analysis of 38 mice from weaning identified the normal survival curve for *Mtm1*^−/y^ mice, with loss of survival starting from 3-4 weeks of age and no mice surviving past 8 weeks of age (Fig. 1A). Analysis of body weight progression in mice showed that the average weight of 3-week-old mice at the start of the study was 10.31±1.27 g (mean±s.d.). In the majority of mice, this increased from weeks 3-4, followed by a decline in body weight, with no mice reaching 20 g in this study (Fig. 1B). Next, a joint model was generated from the raw body weight data, considering both survival and evolution of body weight. Based on this model, the line of best fit and prediction intervals for the expected range of body weights for *Mtm1*^−/y^ mice were modeled (Fig. 1B: black line, gray shadow, respectively).

**Table 1.**
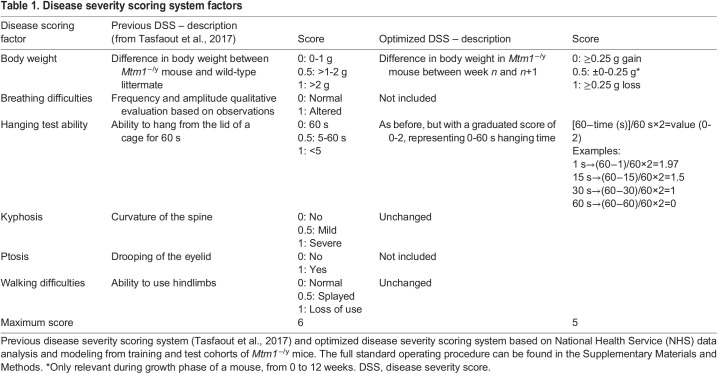
Disease severity scoring system factors

Hanging time upside down from a cage lid with four paws was used as an indicator of whole-body strength in mice, and averaged ∼50 s at 3 weeks of age in *Mtm1*^−/y^ mice (Fig. 1C). From 5 weeks of age, almost no mice could perform the test for more than 5 s, compared to wild-type (WT) mice, which can normally hang for the maximum time tested, 60 s (Koch et al., 2020), at all timepoints analyzed. As statistical modeling for prediction to perform the test in this format was not feasible, we performed a time to event analysis, selecting hanging time ability cutoff times of 50 s (Fig. 1D) or 10 s (Fig. 1E). At 3 weeks of age, ∼60% of *Mtm1*^−/y^ mice could hang for more than 50 s, whereas by 5 weeks of age almost no mice could perform this test for more than 10 s (Fig. 1D,E), indicating rapid and severe progression of the myopathic phenotype during this age bracket.

To capture key disease elements and monitor severity and progression of disease in *Mtm1*^−/y^ mice, previously a disease severity score (DSS) was created (Tasfaout et al., 2017) (Table S1). The DSS was designed to capture key phenotypic elements of the myopathy phenotype apparent in mice. This focused primarily on six key factors: body weight, hanging test (whole-body strength), kyphosis (curvature of the spine), walking ability (hindlimb muscle weakness), ptosis (eyelid muscle weakness) and difficulties in breathing (Table 1). DSS analysis was performed on training cohort data, and modeling was performed to identify the line of best fit and prediction interval of expected scores (Fig. 1F). Mice exhibited an average DSS of 0.88±0.55 (mean±s.d.) at 3 weeks of age, which progressed to 4.44±0.42 (mean±s.d.) by 8 weeks of age, suggestive of a severe disease phenotype in *Mtm1*^−/y^ mice surviving until 8 weeks of age. Overall, analysis of survival and phenotyping data from the training cohort confirm drastically reduced survival and a severe progressive myopathic phenotype in *Mtm1*^−/y^ mice from weaning until 8 weeks of age.

### Natural history study in test cohort of myotubular myopathy mice

Following on from analysis of historical data in the training cohort, we next performed a natural history study in 20 *Mtm1*^−/y^ mice after weaning, designated the ‘test cohort’. Of note, this colony was generated by *in vitro* fertilization (IVF) from samples taken from *Mtm1*^−/y^ mice from the colony used to generate the training cohort. IVF and colony generation were performed in a separate animal housing facility at a different location, thus more accurately representing the comparison of preclinical research experiments by multiple research laboratories in different locations.

**Fig. 2.**
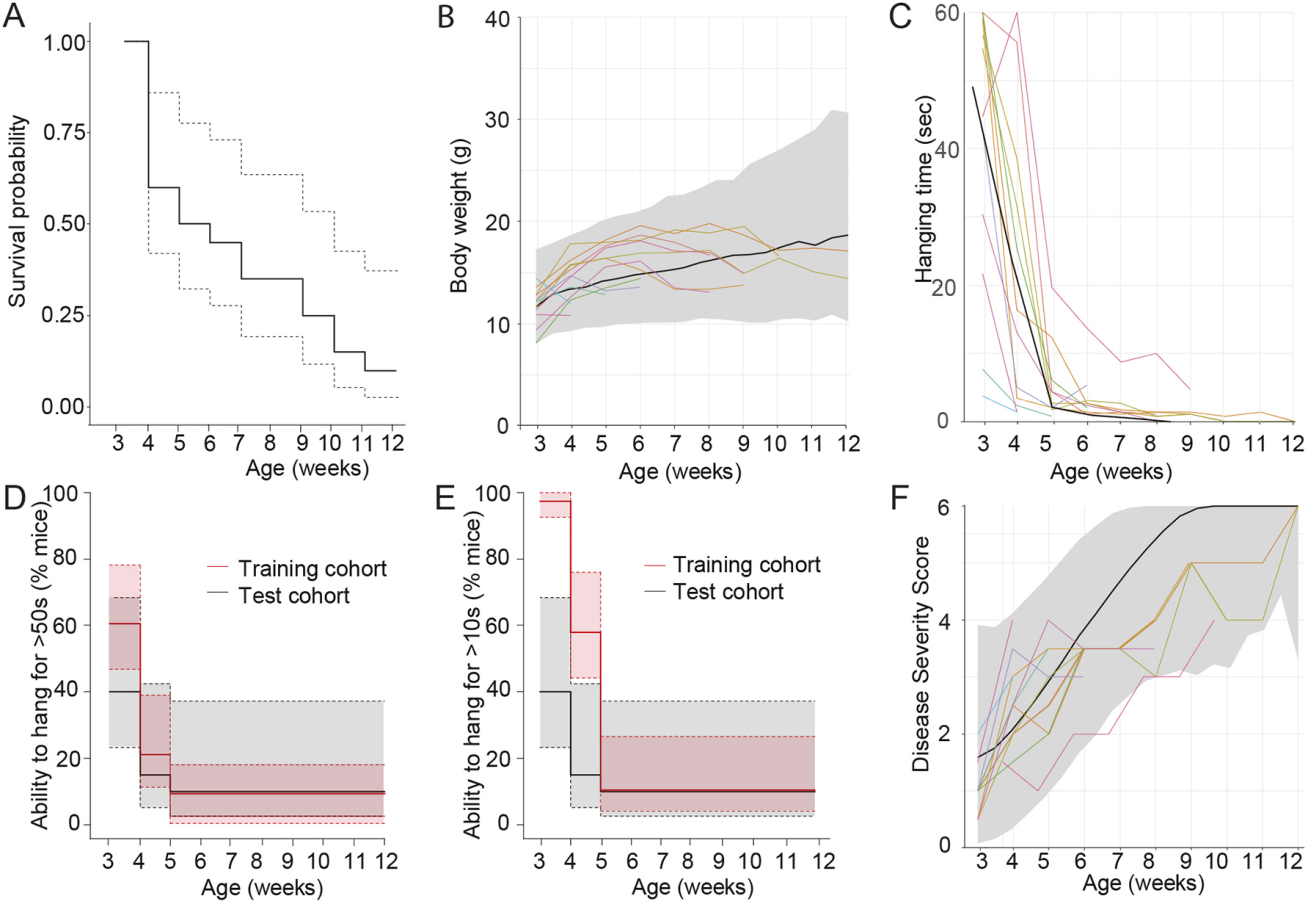
**Natural history study in test cohort of *Mtm1*^−/y^ mice.** (A) Survival of mice, line of best fit (solid line) and 95% CI (dashed lines) are shown. (B) Body weight (g) progression weekly in *Mtm1*^−/y^ mice. Individual mice (colored lines), line of best fit (black line) and prediction interval (shaded gray zone), from the training cohort (Fig. 1B), are highlighted on the graph. (C) Hanging time (60 s maximum), with individual mouse progression shown (colored lines). The line of best fit from the training cohort (Fig. 1C) is highlighted in black. (D,E) Time to event analysis of hanging test, with cutoff times of 50 s (D) and 10 s (E) displayed. Graphs represent line of best fit (solid line) ±95% CI (dashed lines) of training (red, from Fig. 1D,E) and test (black) cohorts for comparison. (F) DSS, with a score between 0 (unaffected) and 6 (most severely affected) awarded per mouse per week, based on six different phenotypes (see Table 1 for details). Line of best fit (black) and prediction intervals (gray shaded area) are shown from the training cohort for reference (Fig. 1F). All data in this figure represent analysis of *Mtm1*^−/y^ mouse phenotypes from the test cohort (or training cohort where indicated), from 3 weeks of age; individual mice represented as colored lines. *n*=20 male mice.

Analysis of survival in the test cohort of *Mtm1*^−/y^ mice was performed. Loss of survival was observed from 3 to 4 weeks of age, and no mice survived past 12 weeks of age (Fig. 2A), suggesting more variation in survival in the test cohort compared to the training cohort (Fig. 1A). Based on the body weight data generated in the training cohort (based on raw data up to 8 weeks of age), a model was generated to display the line of best fit (median profile) and prediction interval expected for body weight progression in *Mtm1*^−/y^ mice (Fig. 1B). Body weight was then analyzed weekly in the test cohort and overlaid on the model generated from training data, including extrapolation of expected prediction interval for 8-12 weeks of age. All mice fell within the expected weight range for *Mtm1*^−/y^ mice over the period analyzed (3-12 weeks of age), confirming the reproducibility of body weight progression in this mouse line (Fig. 2B). Consistent with the training colony, no mice reached 20 g body weight.

To analyze whole-body strength, the hanging test was then performed in the test cohort of *Mtm1*^−/y^ mice. As observed in the training cohort (Fig. 1C), hanging time rapidly declined in mice from 3 to 5 weeks of age (Fig. 2C). A statistical comparison between training and test cohorts was performed using a time to response analysis (Fig. 2D,E), confirming the reliability of this test as an indicator of whole-body strength in *Mtm1*^−/y^ mice.

Finally, the combined DSS was analyzed. Based on the model generated from the training cohort (Fig. 1F), which included extrapolation of expected prediction interval for 8-12 weeks of age, all mice fell within the expected DSS over the period analyzed (Fig. 2F, 3-12 weeks of age), confirming the reproducibility and reliability of DSS progression in this mouse line, and the analytical validity of the model generated.

### Optimized disease severity analysis based on combined natural history study data from training and test cohorts

**Fig. 3.**
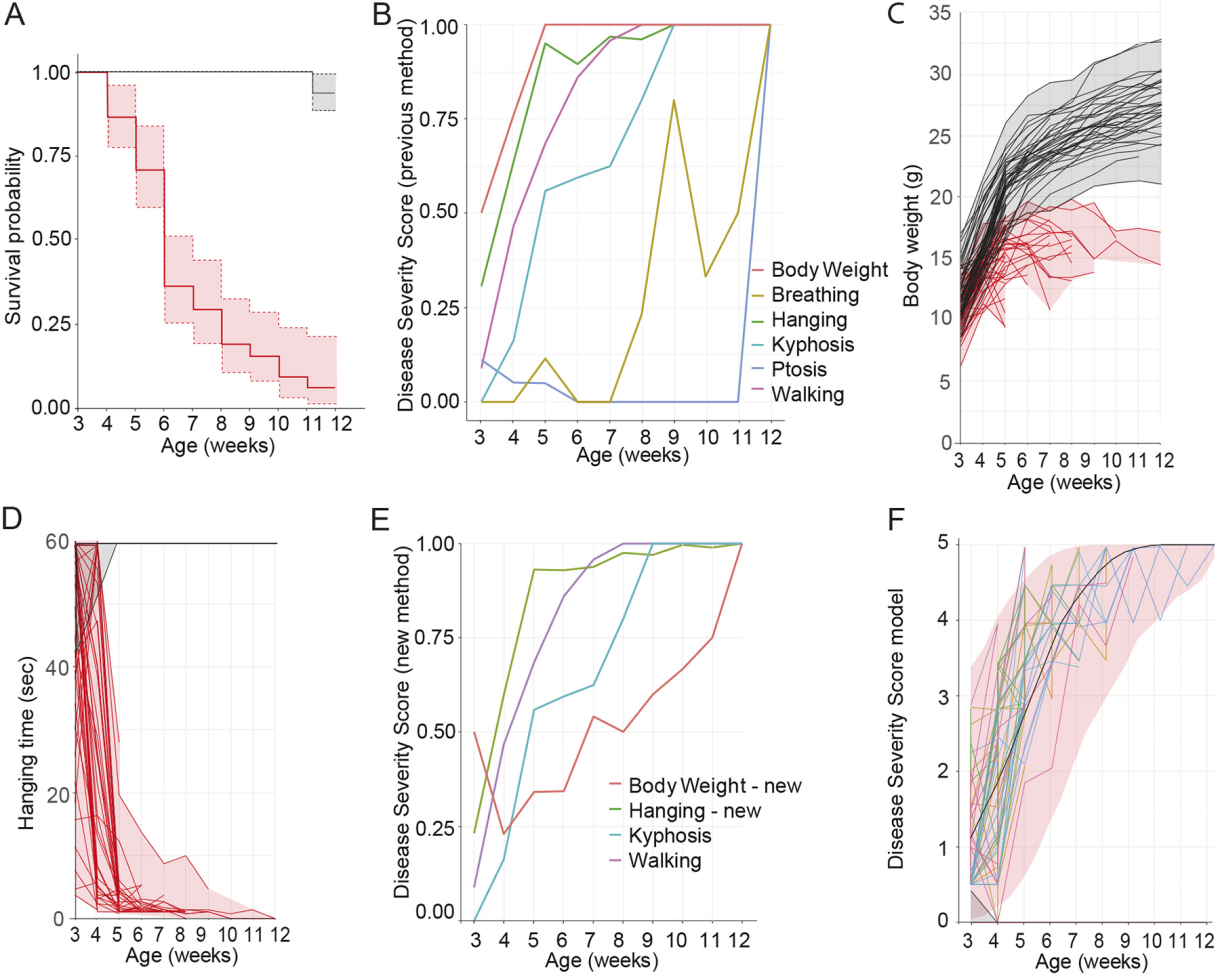
**Optimized disease severity analysis based on combined natural history study data from training and test cohorts.** (A) Survival of mice, line of best fit (solid line) and 95% CI (dashed lines) shown for wild-type (WT; black) and *Mtm1*^−/y^ (red) mice. (B) Line of best fit shown for progression of each of the six individual factors of the DSS for *Mtm1*^−/y^ mice (see Table 1 for details). (C,D) Individual body weight (C) and hanging time (D) from combined natural history study data from training and test cohorts for WT (black) and *Mtm1*^−/y^ (red) mice. (E) Line of best fit shown for progression of each of the selected four individual factors comprising the optimized DSS in *Mtm1*^−/y^ mice (see Table 1 for details). (F) DSS progression of individual *Mtm1*^−/y^ mice, with a score between 0 (unaffected) and 5 (most severely affected) awarded per mouse per week, based on four different phenotypes (see Table 1 for details, hanging test maximal value of 2 possible). Line of best fit (black), and prediction intervals (shaded area) modeling is shown. All WT mice had a score of 0 from week 4 onwards (black lines, shading). All data in this figure represent analysis of WT (*n*=64) and *Mtm1*^−/y^ (*n*=58) mouse phenotypes from the training and test cohorts, from 3 weeks of age; individual mice represented as colored lines.

Based on the large volume of data generated above, we next aimed to optimize the disease severity analysis performed in *Mtm1*^−/y^ mice, by focusing on a statistical modeling approach. First, combined analysis of the survival of *Mtm1*^−/y^ mice highlighted that deaths could occur from the age of weaning at 21 days (youngest age analyzed in this study), until 12 weeks of age, with less than 50% of *Mtm1*^−/y^ mice surviving past 5 weeks of age, compared to 100% of WT mice (Fig. 3A).

Next we investigated the disease severity in the combined training and test cohorts. The DSS focuses on six factors – body weight, hanging test, kyphosis, walking ability, ptosis and difficulties breathing – as described in Table 1. The progression of each of the individual six factors of the combined training and test mouse cohorts is shown in Fig. 3B (average values shown). Although body weight, hanging, walking and kyphosis reflected early disease progression in *Mtm1*^−/y^ mice from 3 to 7 weeks of age, breathing and ptosis did not reflect disease progression, with elevated scores only occurring after 7 weeks of age, when 20% of mice were still alive. Therefore, we decided to focus on the four factors best reflecting early disease progression. Body weight progression in *Mtm1*^−/y^ mice (Fig. 3C, red) clearly identified a pattern of weight gain, not reaching a maximum of 20 g, before a sharp decline in body weight prior to death, compared to WT mice (black), which continued to increase in body weight. On average, body weight decline started 2.3 weeks before death, with an average body weight loss of 2.16±1.07 g. For this reason, the parameters measuring body weight were optimized to reflect body weight gain (0, expected in healthy juvenile mice), stabilization (0.5) or decline (1) relative to the prior week in the same mouse (Table 1), rather than relative to WT mice. Hanging test, reflecting whole-body strength, was identified as the best representative of the myopathic phenotype in *Mtm1*^−/y^ mice, with a rapid decline in performance identified from 3 to 5 weeks of age, compared to WT mice, which could perform the test for the duration of the experiment (Fig. 3D). Consequently, hanging ability was given a higher weight of 2 points. It was also constructed as a continuous score between 0 and 2 instead of the previously used categorical scores of 0, 0.5 and 1 (Table 1; Table S1). The scores for kyphosis and walking were deemed appropriate, and no optimization was required. The line of best fit for each DSS parameter is shown in Fig. 3E, updated based on statistical analysis and modeling of natural history study data from training and test cohorts in mice. This reflected a progressive and severe development of all disease phenotypes analyzed in *Mtm1*^−/y^ mice from 3 to 12 weeks of age. Of note, the two functional tests, hanging ability (whole-body strength) and walking ability, were the first to decline in *Mtm1*^−/y^ mice, followed by physical parameters, kyphosis and finally body weight.

The next step was to combine these factors for an overall DSS, with a maximum value of 5. Individual progression of the optimized DSS is shown for *Mtm1*^−/y^ mice, confirming a rapid disease progression from 3 weeks of age (Fig. 3F). These data were then used to optimize the DSS model, represented on the graph, with the line of best fit and prediction interval shown (Fig. 3F). Not surprisingly, all mice from training and test cohorts fell within the predicted range for almost all timepoints analyzed. A standard operating procedure to perform this optimized DSS can be found in the Supplementary Materials and Methods.

### Investigation of post-mortem muscle phenotypes in *Mtm1*^−/y^ mice

**Fig. 4.**
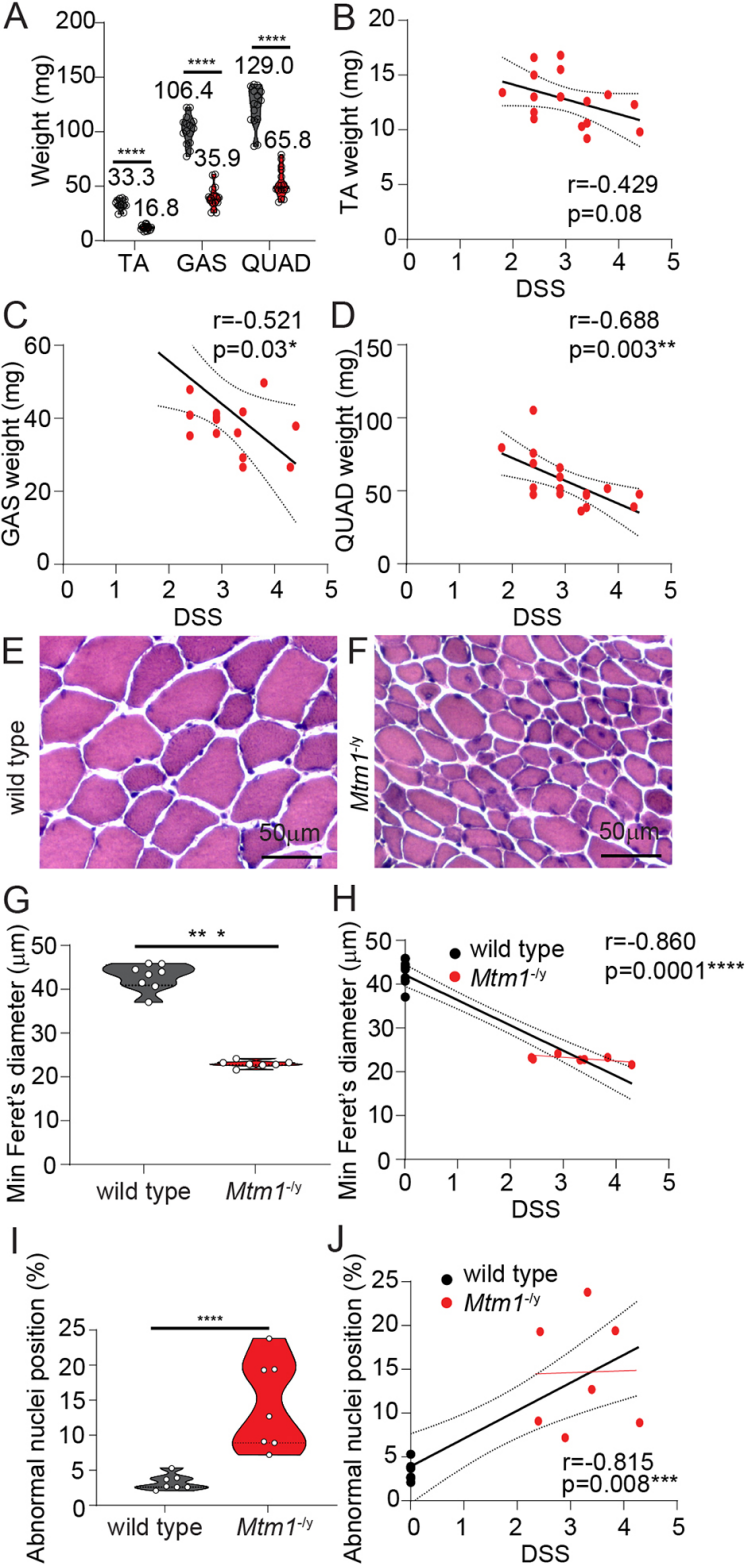
**Skeletal muscle analysis relative to disease severity in *Mtm1*^*−/*y^ mice.** (A) Muscle mass of tibialis anterior (TA), gastrocnemius (GAS) and quadriceps (QUAD) muscles from WT and *Mtm1*^−/y^ mice. (B-D) Correlation analysis of TA (B), GAS (C) and QUAD (D) muscle mass relative to DSS for *Mtm1*^−/y^ mice. Line of best fit and 95% CI are highlighted. Representative Hematoxylin and Eosin (HE) staining of TA muscles from WT (E) and *Mtm1*^−/y^ (F) mice (scale bars: 50 µm). (G,H) Analysis of fiber size, represented as minimum feret's diameter presented alone (G) or relative to DSS (H). (I,J) Analysis of fibers with altered nuclei positioning presented alone (I) or relative to DSS (J). (H,J) Line of best fit and 95% CI highlighted for all mice (black), and line of best for *Mtm1*^−/y^ mice alone (red). (A,G,I) Represented as violin plots, individual mouse data shown. Spearman correlation analysis performed for C,D,H,J; Pearson correlation analysis for B; unpaired *t*-test for A (TA and QUAD),I; unpaired *t*-test with Welsh's correction for A (GAS),G. *P<0.05, **P<0.01, ***P<0.001, ****P<0.0001.

We next wanted to assess whether the disease severity phenotype observed in *Mtm1*^−/y^ mice correlated with muscle pathology. To this end, we examined the muscle mass of several skeletal muscles. Muscle atrophy was similarly observed across tibialis anterior (TA), gastrocnemius and quadriceps skeletal muscles (2- to 3-fold reduction in mass, Fig. 4A; 1.5- to 1.9-fold relative to body weight, Fig. S1A,B). Muscle mass correlated with disease severity in *Mtm1*^−/y^ mice from all skeletal muscles analyzed (Fig. 4B-D). As the name suggests, CNM results in the abnormal positioning of nuclei internally within muscle fibers. Focusing on the TA muscle, fiber diameter and nuclei position were significantly altered in *Mtm1*^−/y^ mice versus WT mice (Fig. 4E-I). Although a significant correlation was observed when analyzing DSS compared to fiber size and nuclei position in WT and *Mtm1*^−/y^ mice, no correlation was observed when analyzing only *Mtm1*^−/y^ mice at 5 weeks of age (Fig. 4H,J). This may be explained by the muscle function parameters of the DSS (walking and hanging ability), which are already nearly maximally affected by this age, whereas the variable components at this age (kyphosis and body weight) are less related to muscle structure and function (Fig. 3E).

### Validation of the model in a dose–response study following DNM2 reduction

**Fig. 5.**
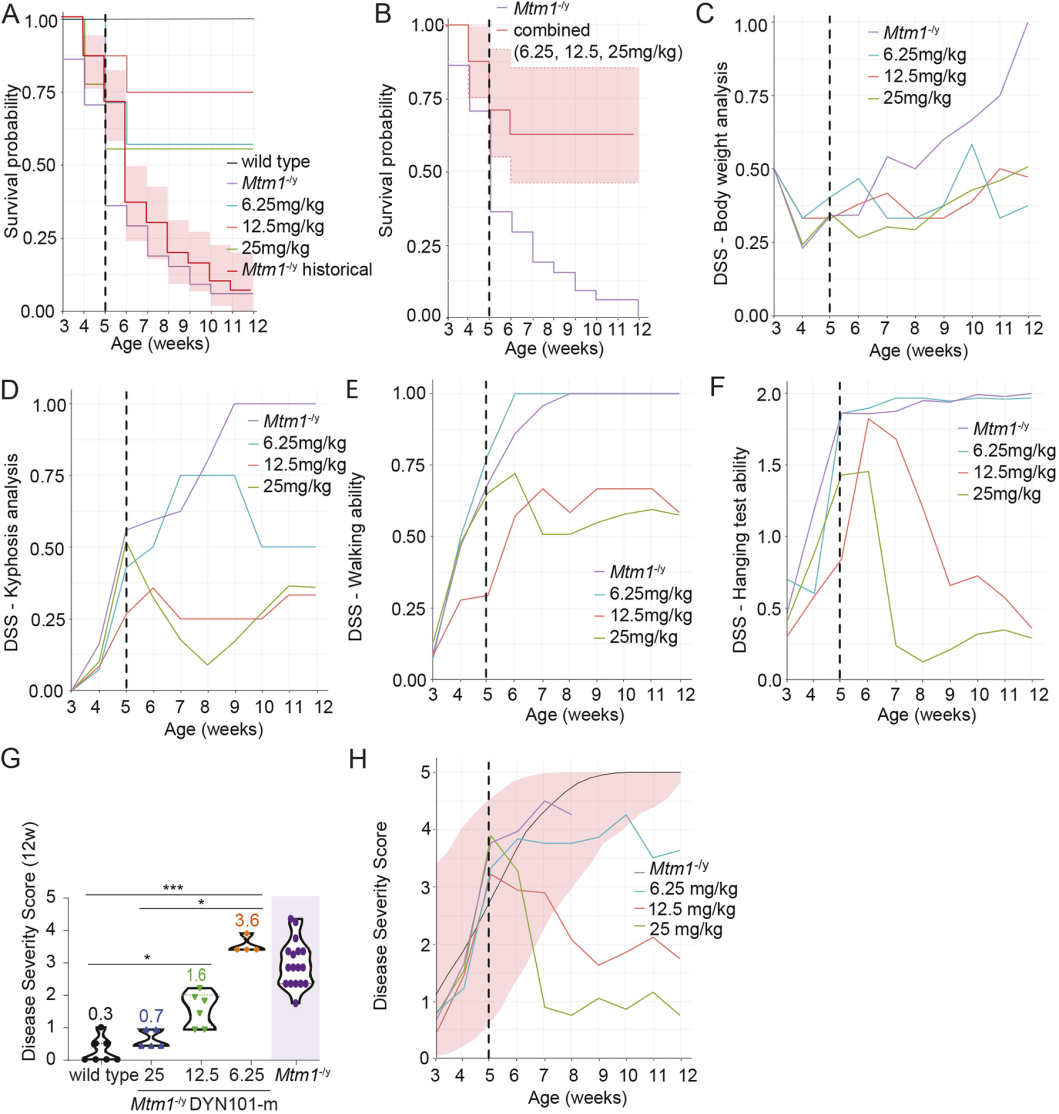
**Validation of the model in a dose–response study with DNM2 therapy.** Three doses were selected: low, mid and high (6.25, 12.5, 25 mg/kg; blue, red, green lines, respectively) doses of DYN101-m targeting murine *Dnm2* were delivered weekly by intraperitoneal injection from 5 to 12 weeks of age to *Mtm1*^−/y^ mice, and compared to WT (black) and *Mtm1*^−/y^ control mice injected with antisense oligonucleotide (ASO) control (purple) (*n*=7 mice/group). (A,B) Survival of mice shown for all groups separately (A) or with DYN101-m-treated groups combined (B). Average (solid line) and 95% CI (dashed lines) are shown for grouped data (B). (C-F) Individual progression (average) of individual DSS factors: body weight analysis (C), kyphosis (D), walking ability (E) and hanging test performance (F). (G) DSS of WT and *Mtm1*^−/y^ mice at 12 weeks of age, with a score between 0 (unaffected) and 5 (most severely affected) awarded per mouse, based on four different phenotypes (C-F). DYN101-m dosing indicated where relevant (mg/kg). DSSs from *Mtm1*^−/y^ mice at 7 weeks of age from Fig. 4 shown for reference only (purple). (H) DSS model (Fig. 3D) represented by line of best fit (black) and prediction intervals (shaded area) modeling is displayed; line of best fit for joint longitudinal survival and phenotyping of *Mtm1*^−/y^ mice (control, low, mid, high dose) in the dose–response study is indicated. Vertical dashed black lines indicate time of first injection. Kruskal–Wallis test performed for G. *P<0.05, ***P<0.001.

The final step to validate the disease severity model generated was to test a therapeutic intervention in *Mtm1*^−/y^ mice. Reduction of DNM2 by systemic delivery of ASOs was shown to rescue myotubular myopathy in 3-week-old *Mtm1*^−/y^ mice in a dose–response manner (Tasfaout et al., 2017; Koch et al., 2020). Here, we tested the dose–response effect of treatment of *Mtm1*^−/y^ mice with ASOs targeting murine *Dnm2*, by weekly intraperitoneal dosing from 5 weeks of age, an age at which mice are already severely affected by the disease (Fig. 3). Low, mid and high (6.25, 12.5, 25 mg/kg, respectively) doses of the 16-nucleotide ASO candidate targeting murine *Dnm2* (DYN101-m), or ASO control (25 mg/kg) (Tasfaout et al., 2017), were delivered weekly by intraperitoneal injection from 5 to 12 weeks of age. A clear increase in survival was observed in *Mtm1*^−/y^ mice at all doses tested (Fig. 5A), compared to untreated *Mtm1*^−/y^ mice in this study. All WT mice survived for the duration of this study. As no dose response was identified for survival in this study, survival data across groups were combined and compared to those of untreated *Mtm1*^−/y^ mice. Combined survival analysis, represented as mean±95% confidence interval (Fig. 5B), was clearly above the survival observed in untreated *Mtm1*^−/y^ mice, indicating that survival significantly increased following DYN101-m administration in this trial.

To investigate the therapeutic effect of DNM2 reduction on disease severity in *Mtm1*^−/y^ mice, we next analyzed each of the four DSS parameters in mice from the dose–response study. Regarding physical parameters, body weight progression (Fig. 5C) and kyphosis (Fig. 5D), a clear improvement was observed in *Mtm1*^−/y^ mice following reduction of DNM2 at all doses at the completion of the study (12 weeks of age). Of note, *Mtm1*^−/y^ mice injected with the low dose (6.25 mg/kg) showed a delayed response for improvement in presentation of kyphosis, compared to mid and high doses (12.5 mg/kg and 15 mg/kg, respectively). A clear improvement was observed in functional parameters, walking (Fig. 5E) and hanging ability (Fig. 5F), at mid and high doses, whereas no improvement was observed in these parameters at low dose, despite the improved survival. Furthermore, a delayed improvement in hanging test ability was observed in mid compared to high dose, suggesting a dose–response effect.

Finally, the combined DSS was analyzed in *Mtm1*^−/y^ mice treated with ASOs targeting *Dnm2*. An improvement in DSS was observed at all dose ranges tested (Fig. 5G), with all doses resulting in mice with reduced DSSs, outside of the predicted range for untreated *Mtm1*^−/y^ mice of the same age (Fig. 5H). Importantly, a dose–response effect was observed, with higher doses resulting in a more rapid improvement of the disease phenotype, thus validating the disease severity model generated, and confirming a reduction of the myopathy features in a dose–response manner following *Dnm2* reduction.

### Histopathological analysis of dose–response study with DNM2 therapy

**Fig. 6.**
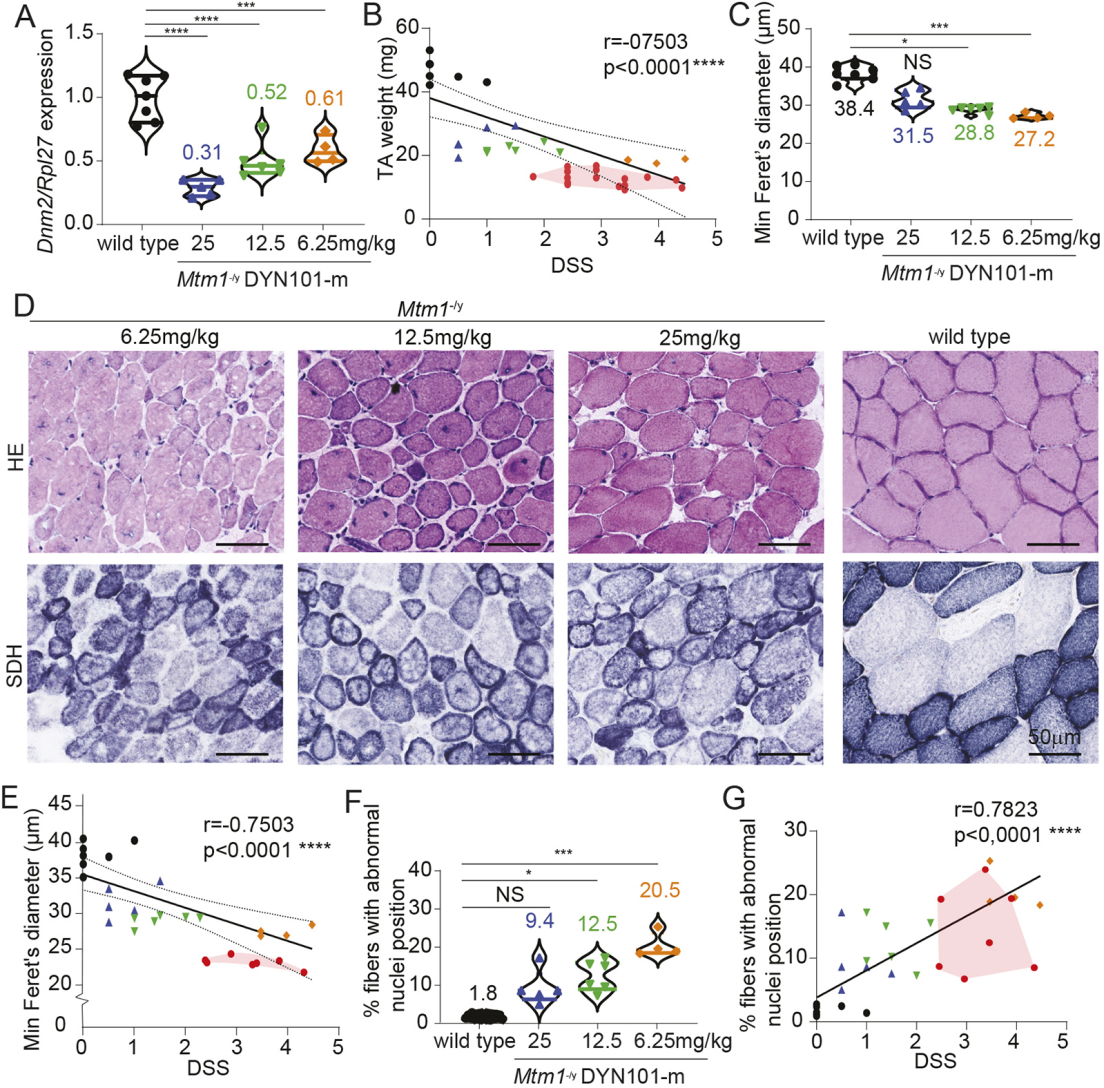
**Correlation analysis of post-mortem skeletal muscle specimens with disease severity following DNM2 therapy.** (A) *Dnm2* mRNA expression quantified by quantitative RT-PCR analysis, relative to *Rpl27* expression, from TA muscles, represented as mean±s.d. Ordinary one-way ANOVA followed by Dunnett's multiple comparisons test performed. (B) TA muscle mass relative to DSS. (C,E) Analysis of fiber size, represented as minimum feret's diameter alone (C) or relative to DSS (E). (D) HE (top row) and succinate dehydrogenase (SDH; bottom row) staining of TA muscles (scale bars: 50 µm). (F,G) Analysis of fibers with altered nuclei positioning displayed alone (F) or relative to DSS (G). (A,C,F) Represented as violin plots; individual mouse data shown, *n*=4-7 mice per group. (C,F) Kruskal–Wallis (non-parametric) followed by Dunn's multiple comparisons test performed. (B,E,G) Spearman correlation tests performed. 12-week-old *Mtm1*^−/y^ mice injected with 6.25, 12.5 or 25 mg/kg are represented by orange, green and blue points, respectively; WT mice are represented by black dots. B, E and G contain as an overlay *Mtm1*^−/y^ mice at 5 weeks of age, reproduced from Fig. 4, purely for comparative purposes, and are not included in statistical analyses here. *P<0.05, ***P<0.001, ****P<0.0001. NS, not significant.

To support the improvement in survival and myopathic phenotype observed following ASO delivery to *Mtm1*^−/y^ mice, we next investigated post-mortem skeletal muscle specimens at the molecular level at 12 weeks of age. Comparison of treated *Mtm1*^−/y^ mice was only possible with WT mice, as untreated *Mtm1*^−/y^ mice do not survive until this age. Following weekly intraperitoneal administration of ASO targeting *Dnm2* into mice, a significant dose-dependent reduction of *Dnm2* mRNA was observed (39-69% reduction, Fig. 6A). Skeletal muscle mass was increased when analyzed alone or relative to body weight in a dose–response manner in both gastrocnemius and TA skeletal muscles (Fig. S1C-F), which correlated with the reduction in DSS observed (Fig. 6B, Fig. 5G; Fig. S1G).

We next investigated whether the reduced myopathic phenotype observed following *Dnm2* reduction correlated with an amelioration of the structural defects observed in *Mtm1*^*−/*y^ mice. Corresponding to the improved whole-body strength observed following *Dnm2* reduction (Fig. 5), an improvement in fiber size was also observed, with no statistical difference observed at the top dose tested compared to that of WT control mice (Fig. 6C,D). Furthermore, this correlated with the improvement in DSS (Fig. 6E). Although mice with the lowest dose still presented with 20.5±3.2% of centralized nuclei, consistent with previously published data in *Mtm1*^−/y^ mice (Tasfaout et al., 2017; Koch et al., 2020), versus 0-2% in WT mice, this was reduced to 9.4±4.6% in the high-dose group, suggesting a dose-dependent improvement in nuclei position in our study (Fig. 6D,F). This was further supported by a clear amelioration of the abnormal organelle accumulations observed by succinate dehydrogenase (SDH) staining of skeletal muscles (Fig. 6D). Importantly, a significant correlation was observed between the DSS and nuclei position across mice and across doses (Fig. 6G). Of note, although an improvement in survival and disease severity was observed in low-dose mice at 12 weeks of age, which was outside of the modeled expected values of *Mtm1*^−/y^ mice at 12 weeks of age (Fig. 5A,H), these mice displayed similar results to untreated *Mtm1*^−/y^ mice at 5 weeks of age (Fig. 6B,E,G, red overlay). Therefore ASO-mediated reduction of *Dnm2* resulted in a significant dose-dependent improvement in disease phenotype, with a clear improvement outside the expected disease progression in untreated *Mtm1*^−/y^ mice.

## DISCUSSION

The purpose of this study was to validate statistically the disease phenotype in *Mtm1*^−/y^ mice and to use these data to generate the first joint longitudinal–survival model of disease progression in this mouse line, which is highly relevant for testing therapeutic approaches preclinically in mice. This study provides an example of optimizing analysis of disease progression and testing therapeutic efficacy in a preclinical model of myotubular myopathy.

### Modeling disease progression in *Mtm1*^−/y^ mice

In this study, we performed disease progression modeling, to provide an optimized model of disease progression in *Mtm1*^−/y^ mice over time based on natural history survival and phenotyping data. The consistency of the disease phenotype between mice from the same colony, and between colonies, is important to understand preclinical disease models. Variability may occur in the phenotype between mice in the same colony, which may affect interpretation of the data generated. Here, we observed minor differences in survival between colonies, which were factored into the survival probability curve generated (Fig. 3A). Survival rates observed in training and test cohorts were consistent with recently published data from the same mouse line housed in three different laboratories (Maani et al., 2018; Gayi et al., 2018; Danièle et al., 2018). Furthermore, both body weight progression and overall disease severity parameters were consistent between colonies (Fig. 2B-F), and used to optimize the model generated for disease severity mapping in *Mtm1*^−/y^ mice (Fig. 3). Of interest, muscle mass correlated with disease severity parameters in 5-week-old *Mtm1*^−/y^ mice; however, no correlation was observed between overall disease severity and muscle histology analysis (Fig. 4), suggesting that muscle histopathology does not reflect the full disease spectrum in this model.

Understanding the natural history of the disease model is the first step in determining study parameters for testing a therapeutic approach, such as timing of the intervention and appropriate parameters to test for therapeutic effects. Here, the data generated were used to select the relevant parameters to analyze disease progression in *Mtm1*^−/y^ mice and improve the disease severity scoring system using statistically powered data. The model developed here from phenotyping of over 50 *Mtm1*^−/y^ mice from two independent colonies suggests a progressive myopathic phenotype in mice, which provides a window for testing therapeutic potential post-weaning (Fig. 3). This statistical model will be made available to any researcher working with this mouse line, which can be of use to validate future therapeutic approaches tested on this mouse line in different laboratories. One strength of this approach is that the data used to generate this model came from two independent colonies housed in independent breeding facilities, thus more faithfully represent variation observed by different research laboratories. This modeling approach suggests a method to validate preclinical phenotyping data and therapeutic efficacy that may be applied by scientists testing therapeutic approaches using other neuromuscular disease models. Sharing of historical phenotyping data and modeling approaches across publicly accessible platforms is an important collaborative step that researchers can contribute to, which can help to increase the reliability of preclinical data generated. Supporting this notion, a second study (Sarikaya et al., 2022) published in this issue focuses on disease progression of an independent cohort of *Mtm1*^*−/*y^ mice on the C57B6/J background. This comprehensive study supports the consistency of the progressive myopathic phenotype observed in this mouse line (motor performance and muscle mass – the latter supported by MRI imaging), whilst highlighting subtle differences likely due to the distinct mouse strains. In addition, the complementary study by Sarikaya and colleagues includes detailed omics analysis, which sheds light on the molecular mechanisms that accompany the phenotyping and histopathological data presented here, and provides insight into the temporal pathogenic increase observed in DNM2 protein expression.

### Use of preclinical models to test therapeutic efficacy

It is of high importance to statistically validate the model(s) used for neuromuscular disorders and analyze therapeutic efficacy in an unbiased manner. We used the model generated here of disease progression in *Mtm1*^*−/*y^ mice to validate whether reducing *Dnm2* expression improves survival and disease phenotype in these mice. Targeting *Dnm2* by ASO-mediated reduction blocked disease progression (low dose) and resulted in a significant reduction in disease phenotype (mid/high dose), with a clear improvement outside the normal range of disease progression in *Mtm1*^*−/*y^ mice in all dose groups (Fig. 5). This study followed on from the initial proof-of-concept study validating targeting *DNM2* with ASOs as a potential target for therapy (Tasfaout et al., 2017) and was carefully designed to test disease reversion in a dose–response study (Figs 5 and 6) (Koch et al., 2020).

Performing statistically powered blinded studies can help improve the correct interpretation of preclinical data, as can reproducing data from more than one colony. Initiatives such as the TREAT-NMD neuromuscular network offer support for preclinical research (https://treat-nmd.org/research-overview/), as well as guidance and advice to scientists on the different aspects of translational research related to therapy development programs in neuromuscular disorders, with the aim of improving translational efficacy for the benefit of patients [TREAT-NMD advisory committee (TACT)] (Willmann et al., 2020). Therapeutic data in mouse models of disease often provide the main or sole data source supporting therapeutic improvement in disease presentation, with the majority of the supportive non-clinical data aimed at reducing any potential safety risk(s) to patients and understanding pharmacokinetics to translate the dose to the human context (Cowling and Thielemans, 2019). Currently three clinical trials (NCT04915846, NCT03199469, NCT04033159, the latter supported by data generated here) have been initiated following therapeutic proof-of-concept observed from three independent therapeutic approaches in this mouse model (Buj-Bello et al., 2008; Buono et al., 2018; Tasfaout et al., 2017; Maani et al., 2018; Gayi et al., 2018). Of note, preliminary therapeutic efficacy has been observed in myotubular myopathy patients following preliminary data generated with the most advanced of the therapeutic approaches (Shieh et al., 2020), supporting the potential utility of *Mtm1*^−/y^ mice as a disease model for relevant myotubular myopathy disease phenotypes in patients.

In conclusion, we present here a statistical modeling approach for disease progression in myotubular myopathy mice and validate the reliability of this model by testing a therapeutic approach in this mouse line in a dose–response study. This approach can be applied by researchers across the neuromuscular field, to support the generation of reliable and reproducible preclinical data. Using this approach may thus improve confidence in preclinical therapeutic data generated from neuromuscular disease models across different laboratories or across different cohorts.

## MATERIALS AND METHODS

### Generation of *Mtm1*^*−/*y^ mice

*Mtm1*^*−/*y^ or WT 129SvPAS mice were previously generated and characterized by crossing *Mtm1* heterozygous females obtained by homologous recombination with WT males (Buj-Bello et al., 2002). The training cohort was previously generated at the Institut de Génétique et de Biologie Moléculaire et Cellulaire (IGBMC; Illkirch, France), and historical published data generated from this cohort were obtained and analyzed from 3 weeks of age [studies 3, 5, *n*=38 *Mtm1*^*−/*y^ mice (Koch et al., 2020)]. To generate the test cohort, IVF was performed in the 129SvPAS strain at Janvier Laboratories (Saint-Berthevin, France), then offspring were transferred to Chronobiotron (Strasbourg, France) for colony generation of the test cohort. Animal experimentation was approved by the institutional ethical committee, the training cohort and ASO administration were approved by the Comité d'Ethique pour l'Expérimentation Animale IGBMC-Institut Clinique de la Souris (APAFIS#5453-2016052510176016 v5), and the test cohort was approved by the Comité Régional d’Ethique en Matière d’Expérimentation Animale de Strasbourg Chronobiotron (20183-2019040817583412 v5 and v6). Test cohort mice were analyzed from 3 weeks of age (*n*=20 *Mtm1*^*−/*y^ mice). For both the training and test cohorts, daily observation was performed when necessary. In the case of severe phenotype, scoring was performed to determine whether action was needed to reduce pain, or whether humane endpoints were reached, in which case the mice were sacrificed. Mice were humanely sacrificed when required according to national and European legislations on animal experimentation. Male mice were analyzed in this study. Animals were housed in a temperature-controlled room (19-22°C) with a 12:12 h light/dark cycle, with free access to food. See Table S2 for additional information.

### ASOs

ASOs were chemically modified with phosphorothioate in the backbone and cEt modifications on the wings with a deoxy gap (3-10-3 design). ASOs were synthesized by IONIS Pharmaceuticals as previously described (Tasfaout et al., 2017). Both the 16-nucleotide ASO candidate targeting murine *Dnm2* (DYN101-m, GGCATAAGGTCACGGA) and the control sequence with no homology to the mouse genome (ASO-Ctrl, GGCCAATACGCCGTCA) were previously validated (Tasfaout et al., 2017; Buono et al., 2018). ASOs were dissolved in filtered and autoclaved sterile D-PBS (Life Technologies, #14190-144). Intraperitoneal injections of 6.25, 12.5 or 25 mg/kg of ASO were performed in *Mtm1*^*−/*y^ or WT male mice weekly, from 5 to 12 weeks of age (*n*=7 mice/group). Mice were sacrificed 2 days after the final injection (Koch et al., 2020).

### Generation of mouse cohorts

Training cohort data were generated from historical data of *Mtm1*^*−/*y^ mice located at the IGBMC animal facility (Koch et al., 2020). The test cohort was derived as follows: IVF was performed (Janvier Laboratories, Rennes, France) with samples taken from *Mtm1*^−/y^ mice from the colony used to generate the training cohort, colony generation was performed, and then the animals were transferred to Chronobiotron animal facility for colony amplification and phenotyping.

### DSS analysis

DSS was performed to monitor the clinical appearance of *Mtm1*^−/y^ mice. The DSS was designed to evaluate the clinical evolution of six indicators of myopathy in mice: body weight difference, hanging test ability, kyphosis, hindlimb position whilst walking, breathing ability and ptosis, as described previously (Tasfaout et al., 2017). Details of DSS analysis, both previous and updated method based on analyses performed in this study, are represented in Table 1.

### Hanging test

Mice were placed on a grid (cage lid, dimensions 410×270 mm), which was then inverted and held 40 cm above the cage litter; the latency to fall was measured three times for each mouse, with a minimum of 5 min interval between trials to allow a recovery period. The latency time measurements began from the point when the mouse was hanging free on the grid and ended with the animal falling to the cage underneath the grid. The maximum time measured was 60 s. Results are expressed as an average of three trials.

### Statistics and modeling

For the ‘training cohort’, historical data were accessed, where the sample size (*n*) was established for each individual study (Koch et al., 2020). For the ‘test cohort’, we calculated the statistical power using R software function ‘pwr2::ss.1way’ (k, number of groups; alpha; beta; f, effect size; delta, smallest difference among k group; sigma, s.d., i.e. square root of variance; and B, iteration times). The statistical power was calculated as *n*=15 for the ‘test cohort’ to see a difference between groups; however, *n*=20 was used to optimally model the progression of the disease in *Mtm1*^−/y^ mice. For the ‘dose–response’ ASO study, the prespecified effect size was established to be *n*=7 (Tasfaout et al., 2017).

To model body weight progression, a joint model considering survival and body weight progression was used. The evolution was not linear with time but rather changed, as the square root of weeks and the log weight are modeled in order to account for the natural lower bound at 0. Random slopes and intercept for each mouse are included. See Supplementary Materials and Methods for a more detailed description of the joint models, including for DSSs. Correlation analyses were performed with a Spearman or Pearson correlation test, as noted in the figure legends. Additional statistical analyses were performed as stated in the figure legend where appropriate, with *P*<0.05 considered significant. The normality of the residuals and the variance homogeneity were assessed to apply the appropriate statistical test with GraphPad Prism 9.

### Exclusion criteria

All exclusion/inclusion criteria were pre-established. All male mice from 3 weeks of age were included. For the dose–response study, mice entered the study at 3 weeks of age; however, mice that died or were sacrificed for humane endpoints before starting the injection protocol at 5 weeks of age were excluded and replaced. For sample analysis, the exclusion criteria were (1) technical issues not allowing the data to be measured, or (2) sample(s) detected as an outlier with GraphPad Prism 9 (parameters: ROUT, Q=5%).

### Randomization and blinding

For the dose–response experiment, *Mtm1*^*−/*y^ or WT mice were randomly assigned to groups at 3 weeks of age (*n*=7 mice per group). *Mtm1*^*−/*y^ or WT mice from the same litter were kept in the same cage. Where possible, mice from the same litter were allocated to different dosing groups. For the histological analyses in the dose–response study, the ASO dose administered was blinded to avoid any bias.

### Quantitative RT-PCR

RNA was isolated from organs using a NucleoSpin RNA kit (Macherey Nagel). For muscle tissue, the supplementary protocol available from the manufacturer entitled ‘isolation of RNA from fibrous tissue’ (https://www.mn-net.com/fr/nucleospin-rna-mini-kit-for-rna-purification-740955.50) was used. Reverse transcription was carried out on 250 ng aliquots using SuperScript™ IV Reverse Transcriptase (ThermoFisher Scientific). Quantitative PCR was done with PowerUp™ SYBR™ Green Master Mix (ThermoFisher Scientific) in a Quant Studio 3 Real-Time PCR System (ThermoFisher Scientific). The relative expression of *Dnm2* mRNA was normalized to *Rpl27*. Primers used were as follows: *Rpl27* Forward, 5′-AAGCCGTCATCGTGAAGAACA-3′; *Rpl27* Reverse, 5′-CTTGATCTTGGATCGCTTGGC-3′; *Dnm2* Forward, 5′-ACCCCACACTTGCAGAAAAC-3′; *Dnm2* Reverse, 5′-CGCTTCTCAAAGTCCACTCC-3′. mRNA analysis followed most Minimum Information for Publication of Quantitative Real-Time PCR Experiments (MIQE) standards.

### Histological analysis of skeletal muscle

Air-dried transverse cryosections (8 μm) were fixed and stained with Hematoxylin and Eosin (HE) or SDH, and image acquisition performed with a slide scanner NanoZoomer 2 HT equipped with brightfield and the fluorescence module L11600-21 (Hamamatsu Photonics). Minimum feret's diameter was analyzed in wheat germ agglutinin (WGA) sections from TA mouse skeletal muscle, using a plugin developed in ImageJ – ‘MyoMage’. Minimum feret's diameter was calculated in >500 fibers per mouse. The percentage of TA muscle fibers with centralized or internalized nuclei was counted in >500 fibers using the cell counter plugin in ImageJ (http://rsb.info.nih.gov/ij/) or FIJI analysis software. Qualitative SDH staining analysis was performed. Fiber to fiber variation in intensity in SDH staining is normal and is indicative of the oxidative state of the fiber. SDH staining is normally relatively homogeneous within each individual fiber. Accumulation within the center or the periphery of a fiber of SDH staining indicates an abnormal distribution.

## Supplementary Material

Supplementary information
