# Supplementary material for: Natural history study and statistical modeling of disease progression in a preclinical model of myotubular myopathy
Source: Dis Model Mech. 2022 Jul 25;15(7):dmm049284. doi: 10.1242/dmm.049284 (PMC9346515; doi:10.1242/dmm.049284)
Supplement: Supplementary information [file dmm-15-049284-s1.pdf]

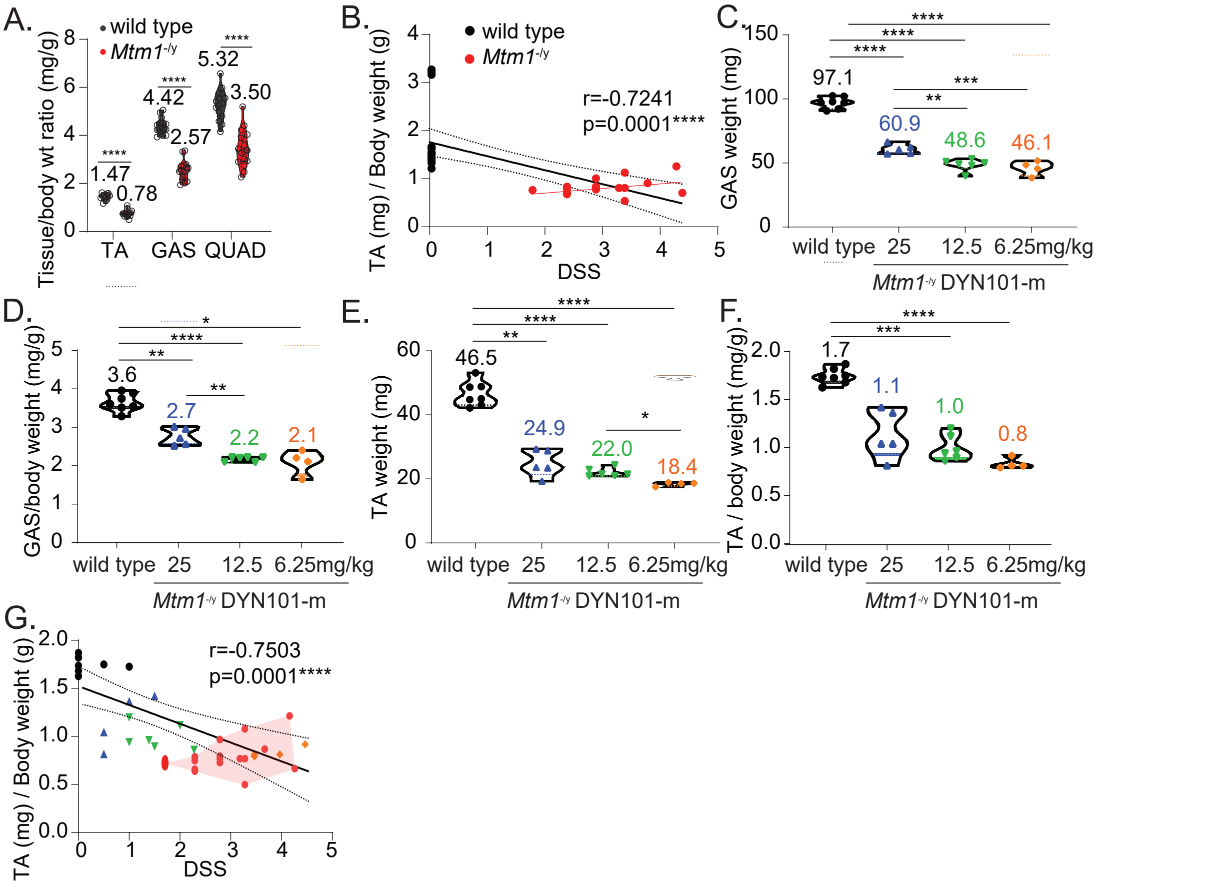

Fig. S1. Analysis of post mortem skeletal muscle speci-mens and correlation with disease severity

(A) Muscle mass of tibialis anterior (TA), gastrocnemius (GAS) and quadriceps (QUAD) muscles relative to body weight, from 5 week old wildtype and *Mtm1*$^{-/y}$ mice. Unpaired t-test with Welsh's correction performed. (B) Correlation analysis of TA muscle mass/body weight ratio, relative to disease severity score (DSS) for 5 week old *Mtm1*$^{-/y}$ (red) and wild type mice (black). Line of best fit and 95%CI highlighted in black for all mice (r= -0.7241; p < 0.0001), line of best fit for *Mtm1*$^{-/y}$ alone (red line). Muscle mass presented alone (GAS-C; TA-E) or relative to body weight (GAS-D; TA-F), for wildtype and *Mtm1*$^{-/y}$ mice injected with 6.25, 12.5 or 25mg/kg DYN101-m targeting murine *Dnm2* reduction. (G) TA/ body weight ratio represented relative to DSS for wild type (black), *Mtm1*$^{-/y}$ mice injected with 6.25 (orange), 12.5 (green), 25mg/kg (blue) DYN101-m. 5 week old *Mtm1*$^{-/y}$ mice from (B) reproduced here for comparison purposes only (red dots, shading), and are not included in statistical analysis. Data represented as a violin plots (A,C-F), individual mouse data shown. Statistical analysis: (A) Unpaired t-test with Welsh's correction; Spearman correlation tests performed for (B), (G) Ordinary 1-way ANOVA followed by Dunnett's multiple comparisons test performed for (C)-(F). *p<0.05, **p<0.01, ***p<0.001,, ****p<0.0001.

Table S1. Previous disease severity scoring system (Tasfaout, Buono et al. 2017). A scoring system was set up to evaluate the clinical evolution of six centronuclear myopathy features. Difference of body weight between *Mtm1*$^{-/y}$ versus WT littermate, ability to perform the hanging test, walking manner, presence or absence of ptosis and kyphosis and breathing difficulties (frequency and amplitude evaluation based on clinical observations) are recorded and a score of 0, 0.5 or 1 is given to each clinical readout. The sum represents the DSS. The higher the DSS, the more severe the phenotype, minimum 0 (healthy mouse), maximum 6 (severely affected mouse) (Tasfaout, Buono et al., 2017).

Disease scoring category	DSS Description	Score
**Body weight**	Score 0-1<br>Difference in body weight between *Mtm1*$^{-/y}$ mouse and wildtype littermate	0: 0-1g<br>0.5: >1-2g<br>1: >2g
**Ptosis**	Score 0 or 1<br>Drooping of the eyelid	0 : No<br>1 : Yes<br>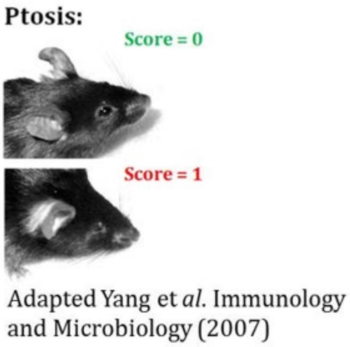
**Hanging test ability**	Score of 0-1, representing 0-60 seconds hanging time.	0: 60s<br>0.5: 5-60s<br>1: <5

Disease Models & Mechanisms • Supplementary information

**Kyphosis**	Score 0-1<br>Curvature of the spine	Score 0: no curvature of the spine<br>Score 0.5: mild curvature of the spine<br>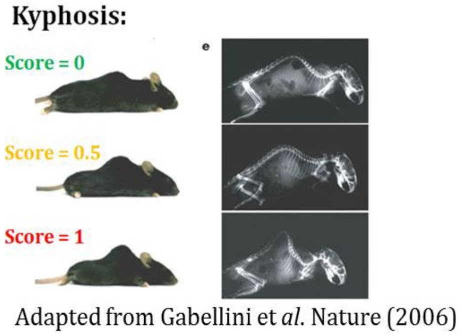<br>Score 1: severe curvature of the spine
**Breathing difficulties**	Score 0 or 1<br>Frequency and amplitude evaluation based on clinical observations	0: no breathing alternation<br>1: breathing alternation
**Walking difficulties**	Score 0-1<br>Ability to use hindlimbs	Score 0: normal use of hindlimbs<br>Score 0.5: splayed use of hindlimbs<br>Score 1: loss of use of hindlimbs<br>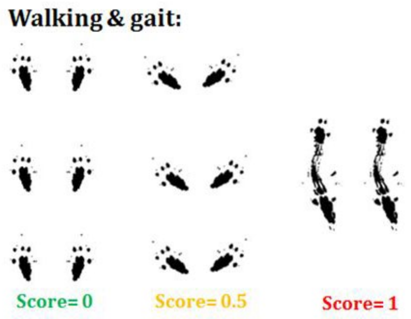
**Maximum score**	6	

Disease Models & Mechanisms • Supplementary information

Table S2. Husbandry and housing conditions for training and test cohorts

	TRAINING COHORT	TEST COHORT
	Training cohort data was generated from historical data of *Mtm1*$^{-/y}$ mice<br>located at the IGBMC animal facility, Illkirch, France (Koch, Buono et al. 2020). N=38 mice	Test cohort data: In vitro fertilization (IVF) was performed (Janvier Laboratories, Rennes, France) with samples taken from *Mtm1*$^{-/y}$ mice from the colony used to generate the training cohort.<br>Following IVF and colony generation, mice were transferred to Chronobiotron animal facility (Strasbourg, France), for colony amplification and phenotyping (test cohort).<br>N=20 mice
**Husbandry conditions**	Light cycle: 12hr/12hr<br>Temperature: 19-22°C<br>Enrichment: nests to help nesting.	<u>Janvier Laboratories :</u><br>Light cycle: 12hr/12hr<br>Temperature: +21°C ± 2°C<br>Enrichment: all cages contain sticks to gnaw (Aspenbrick).<br>Breeding cages were systematically enriched with nests.
**Housing conditions**	Light cycle: 12hr/12hr<br>Temperature: 19-22°C<br>Enrichment: nests	<u>Chronobiotron animal facility:</u><br>Light cycle: 12hr/12hr<br>Temperature: +21°C ± 2°C<br>Enrichment: nests and cardboard tunnel
**Weaning age**	3 weeks of age	3 weeks of age
**Description of cage**	*<u>Cage type 2:</u>*<br>Individually ventilated cages: no<br>Floor surface: 370 cm²<br><br>*<u>Cage type 3:</u>*<br>For housing during experimental procedure, cage type 3 was rarely used because of the larger size and increased distance to access food<br><br><u>Access to food and water:</u><br>Gel diet was added inside the cage to facilitate access.<br>Food pellets were added at multiple places inside the cages.	<u>Chronobiotron animal facility:</u><br>*<u>Cage type 2:</u>*<br>Individually ventilated cages: no<br>Brand: Tecniplast (#1264C Eurostandard) or Erhet<br>Floor surface: 370 cm²<br>Dimensions: L x W x H : 268 x 215 x 141 mm<br>*<u>Cage type 3:</u>*<br>For housing during experimental procedure, cage type 3 was rarely used because of the larger size and increased distance to access to the food.<br>Individually ventilated cages: no<br>Brand: Zoonlab<br>Floor surface: 830 cm²<br>Dimensions: L x W x H : 425 x 265 x 150 mm<br><br><u>Access to food and water:</u><br>Gel diet was added inside the cage to facilitate access.<br>Food pellets were added at multiple places inside the cages.<br>Longer nozzles on water bottles were used to reduce the distance between floor and bottle nozzle.
**Animal density**	4 animals per cage: type II cage<br>5 animal per cages: type III cage	4 animals per cage: type II cage<br>5 animal per cages: type III cage
**Genotype ratio**	Typically 1 or 2 wildtype mice were housed in each cage with *Mtm1*$^{-/y}$ mice from the same litter, whenever feasible.	
**Change frequency**	Once per week	

**Diet**	**Food composition:**	**SAFE® gel diet breeding**	**SAFE® gel diet water**
	Cereals	17.3%	
	Proteins	6.6%	
	Vitamins, minerals	1.2%	
	Fibers	1.4%	1.8%
	Water	72.9%	98.2%

Supplementary Materials and Methods

Joint Models for Disease Severity Scores. Joint models for disease severity scores are presented in the associated manuscript. The mice where followed over a period $[0; \tau\ [$. For each subject i, we observe the following:

- Longitudinal measures: $\{Y_{ij}; j = 1, \dots, n_i\}$ at times $\{t_{ij}\ ; j = 1, \dots, n_i\}$
- Survival time and indicator:

$$T = \min\,(ST, C) \text{ and } \delta = I(ST \le C) = \begin{cases} 1\ if\ uncensored\ observation \\ 0\ if\ censored\ observation \end{cases}$$

The longitudinal measurements and time to event were jointly measured via a latent bivariate process, which was realized independently in each subject $W_i = \{W_{1i_i}, W_{2i}\}$

The longitudinal sub-model is defined as: The observed response y_{ij} scaled between 0 and 1 ($i = 1, \dots, M\ and\ j = 1, \dots, n_i$), is defined as :$y_{ij} \sim Beta\left(a_{ij}, b_{ij}\right)$; where the beta distribution is defined by the parameters a_{ij} and b_{ij} , which are defined, respectively, by the mean μ_{ij} and "the sample size" ν of the distribution as follows:

$$a_{ij} = \mu_{ij} * \nu$$

$$b_{ij} = \left(1 - \mu_{ij}\right) * \nu$$

The parameter ν is estimated from the data, and the mean μ_{ij} is defined as a mixed model with logit-link function, where T is a constant to center time:

$$\chi_{ij} = \alpha_i + \beta * (T + t_{ij})$$

$$\mu_{ij} = \frac{1}{1 + exp\ (-\chi_{ij})}$$

The equation of the mean contains a random effect on the intercept $W_{1i} = \alpha_i \sim N(0, \sigma_\alpha^2)$. In turn, the survival sub-model is then defined via a Weibull survival model:

$$S\left(t_{ij}\right) = \exp\left(-\left(\zeta_i * t_{ij}\right)^k\right)$$

Where: $\zeta_i = \exp(-W_{2i})$

$$W_{2i} = \gamma * \alpha_i$$

Where γ expresses the induced association. A negative value of gamma implies that the larger value of the DSS Score the smaller the probability to stay in the study. A positive value would however imply the opposite. For simplicity in estimation, k = 1 in all estimations of the model indicating a constant failure rate. The model for the evolution of weight over time is similar to the one proposed here over.

The main difference lies in the equation for the mean. Indeed, the equation is assumed to be a linear gaussian model that evolves a the square root of time rather than a beta model.

The disease severity data from individual mice are included only until the point of death. The death of the mouse is then factored into the joint longitudinal survival model for disease severity scores. The above model used here allows the expected range to be presented following the correction for limited sample size, variability, and survival differences. The output is presented as the line of best fit (black line) and prediction intervals (shaded zone). A correct model fit occurs when 95% of the observed individual data is contained, for each time point, in the intervals depicted. Individual data from all living mice is shown as an overlay of the model to confirm this point on each graph, and thus the validity of the model.

Disease Models & Mechanisms • Supplementary information

Standard Operating Procedure for Disease Severity Score

This SOP describes the disease severity scoring (DSS) system, based on the original (DSS) published in Tasfaout, Buono et al 2017, and subsequently optimized in Buono et al (associated manuscript).

Objective

The goal of this SOP is to detail the procedure of allocating a disease severity score (DSS) in mice. The DSS is a parameter which is used for the evaluation of the disease severity of the myopathic phenotype in *Mtm1*$^{-/y}$ mice. The calculation is based on 4 parameters: difference of body weight, hanging test ability, kyphosis and walking difficulties. The minimal score is 0 (no myopathic phenotype) and the maximal score is 5 (characterizing a very severe myopathic phenotype).

1. Abbreviations and Definitions

DSS	Disease Severity Score
NHS	Natural History Study
SOP	Standard Operating Protocol
NA	Not applicable

2. Risk Assessment / Related documents

3. Ethics

Mice should be handled according to national legislation on animal care and experimentation. This SOP is in alignment with the French and European legislation. Procedures and Protocols should be approved by the institutional Ethics Committee.

4. Materials and Equipment

- Type 3 cage: 425 x 265 x 150 mm
- Metal Grid : 410 x 270 mm
- Timer
- Scale
- Lid of transport box (optional)

5. Reagents

NA

Disease Models & Mechanisms • Supplementary information

6. Protocol

1) Body weight

Measure body weight, to 2 decimal places. The score for this parameter (score between 0 and 1) is the difference of a *Mtm1*$^{-/y}$ mouse body weight from week n to n+1:

Table 1: Body weight to score conversion

Body weight	SCORE
**X ≥ 0.25g**	0
**-0.25g > X > 0.25g**	0.5
**X ≤ -0.25g**	1

X(g) = (body weight week n+1(g)) – (body weight week n(g))

2) Hanging test ability

This test must be done one mouse at a time:

a. Take one type 3 cage and one grid.
b. Place mouse in the middle of the grid
c. Turn the grid upside down. The suspending animal should hold on to the grid in order to avoid falling.
   - Test must be set up at a certain height, around 40 cm, for mouse to not being influenced to jump.
d. Prevent the mouse from turning over to the other side of the grid or at pellets food and bottle place by barring with the hand (without touching the mouse) or by using the lid of a transport box.
e. The latency to fall will be measured three times (60 seconds each) for each mouse, with a minimum interval of 10 minutes between trials.
   - The latency time measurements begin when the mouse is hanging free on the wire and end with the animal falling to the cage underneath the wire or grid.
   - If performing the whole body hanging test for the first time, mouse can fall as soon as grip is turned upside down. If this is the case, the first assay can be considered as familiarization of the mouse with the testing conditions and will not be considered as one of the three assays of the test. Only time (seconds) for the three next trials will be reported in the dedicated table (Table 2).
   - If a mouse falls for any other reason that muscle strength default (eg if a mouse is not willing to do the test, voluntary jump from the grip, or falls because of your hands…), this will not be considered as a trial. This must be recorded in the note section in the dedicated table (Table 2)
   - A mouse should normally explore the grid. If a mouse stays in place and does not explore the grip. This must be recorded in the note section in the dedicated table (Table 2)
f. Time (seconds) when mouse falls should be reported in the dedicated table (Table 2).
   - 60 seconds is the maximum time allowed

Disease Models & Mechanisms • Supplementary information

Table 2: Data collection table for hanging test

			Hanging time (seconds)			
**Mice ID number**	**Mice genotype**	**Age (weeks)**	**Test 1**	**Test 2**	**Test 3**	**Note**

Note: Mouse genotype should be blinded until experiment is complete

The score for this parameter (score between 0 and 2) represents the mean hanging time according to the formula: Time (s) > [(60-Time (s))/60s]*2 = value (0-2)

<u>Examples:</u>
1s > (60-1)/60*2=1.97
15s > (60-15)/60*2=1.5
30s > (60-30)/60*2=1
60s > (60-60)/60*2=0

3) Kyphosis

This parameter reflects the curvature of the spine. The score is noted as:

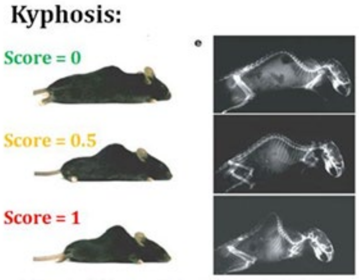

Adapted from Gabellini et *al.* Nature (2006)

Score 0: no curvature of the spine
Score 0.5: mild curvature of the spine
Score 1: severe curvature of the spine

Disease Models & Mechanisms • Supplementary information

4) Walking difficulties

This parameter reflects the ability to use hindlimbs and the ability of mice for walking. The score is noted as:

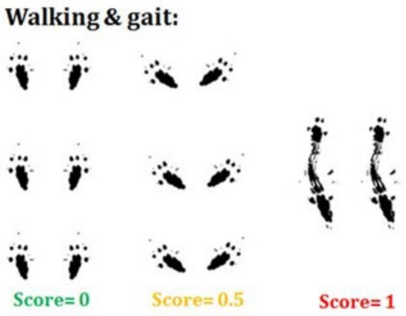

Score 0: normal use of hindlimbs
Score 0.5: splayed use of hindlimbs
Score 1: loss of use of hindlimbs

Note: if score 1:

- indicate if the hindlimbs are paralyzed or not
- indicate if the mouse can still move

7. Calculation of DSS

Disease Severity Score (DSS) is the sum of the scores of the 4 parameters: body weight, hanging test ability, kyphosis and walking difficulties. The maximal DSS is 5.

Note: always refer to ethics application for humane endpoints.

8. Data Collection

All the data concerning the DSS will be reported in the dedicated table (table 3).

Table 3: Data collection table for Disease Severity Score

Mice ID	Mice genotype	Age (weeks)	Body weight	Score body weight	Score hanging	Score walking	Score kyphosis	TOTAL DSS

Note: Mouse genotype should be blinded until experiment is complete

9. Data Analysis

The data will be expressed as a sum of the 4 different parameters: body weight, hanging test ability, kyphosis and walking difficulties

Disease Models & Mechanisms • Supplementary information

Table 4. Disease severity score (DSS). Tabular summary of DSS procedure, based on natural history study data analysis and modeling from training and test cohorts of *Mtm1*$^{-/y}$ mice (Buono et al, associated manuscript).

Disease severity score	*DSS Description*	*Score*
Body weight	Score 0-1<br>Difference in body weight between *Mtm1*$^{-/y}$ mouse from week n to n+1.	Score 0: x ≥ 0.25g<br>Score 0.5: -0.25 > x > 0.25<br>Score 1: x ≤ -0.25g<br>With x being the weight difference
Hanging time	Score of 0-2, representing 0-60 seconds hanging time.	Time (secs) > [(60-Time (s))/60s]*2 = value (0-2)<br><br><u>Examples:</u><br>1s > (60-1)/60*2=1.967<br>15s > (60-15)/60*2=1.5<br>30s > (60-30)/60*2=1<br>60s > (60-60)/60*2=0
Kyphosis	Score 0-1<br>Curvature of the spine	Score 0: no curvature of the spine<br>Score 0.5: mild curvature of the spine<br>Score 1: severe curvature of the spine<br>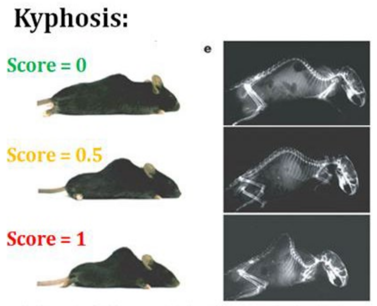<br>Adapted from Gabellini et *al.* Nature (2006)
Walking difficulties	Score 0-1<br>Ability to use hindlimbs	Score 0: normal use of hindlimbs<br>Score 0.5: splayed use of hindlimbs<br>Score 1: loss of use of hindlimbs<br>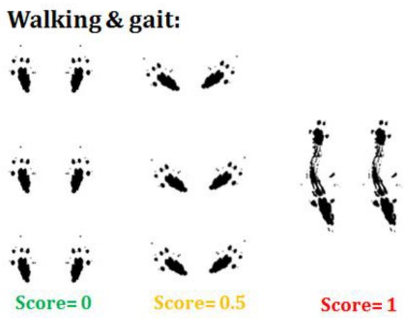
Maximum score	5	

References

Koch, C., S. Buono, A. Menuet, A. Robe, S. Djeddi, C. Kretz, R. Gomez-Oca, M. Depla, A. Monseur, L. Thielemans, L. Servais, C. N. M. S. G. NatHis, J. Laporte and B. S. Cowling (2020). "Myostatin: a Circulating Biomarker Correlating with Disease in Myotubular Myopathy Mice and Patients." Mol Ther Methods Clin Dev **17**: 1178-1189.

Tasfaout, H., S. Buono, S. Guo, C. Kretz, N. Messaddeq, S. Booten, S. Greenlee, B. P. Monia, B. S. Cowling and J. Laporte (2017). "Antisense oligonucleotide-mediated Dnm2 knockdown prevents and reverts myotubular myopathy in mice." Nat Commun **8**: 15661.

Disease Models & Mechanisms • Supplementary information
